# Association between maternal lipid profiles and vitamin D status in second trimester and risk of LGA or SGA: a retrospective study

**DOI:** 10.3389/fendo.2024.1297373

**Published:** 2024-07-01

**Authors:** Xianhua Zheng, Kefeng Lai, Chengyi Liu, Yuan Chen, Xiaodan Zhang, Weixiang Wu, Mingyong Luo, Chunming Gu

**Affiliations:** Department of Clinical Laboratory, Guangdong Women and Children Hospital, Guangzhou, China

**Keywords:** pregnancy, lipid profile, vitamin D, large for gestational age, small for gestational age

## Abstract

**Background:**

Accumulating evidence has linked dyslipidemia during pregnancy to the risk of delivering infants born either large for gestational age (LGA) or small for gestational age (SGA). However, the effects of the vitamin D status on these relationships require further investigation. This study investigated whether the relationship between lipid profiles and the risk of LGA or SGA was influenced by vitamin D levels during the second trimester.

**Methods:**

Maternal lipid profile levels, including total cholesterol (TC), triglyceride (TG), low-density lipoprotein cholesterol (LDL-C), high-density lipoprotein cholesterol (HDL-C), and vitamin D levels, were measured in a cohort of 6,499 pregnant women during the second trimester. Multivariate regression models and subgroup analyses were employed to evaluate the potential associations between maternal lipid profiles, vitamin D levels, and the risk of LGA or SGA.

**Results:**

The prevalence of SGA infants was 9.8% (n=635), whereas that of LGA infants was 6.9% (n=447). Maternal TG levels were found to be positively associated with the risk of LGA (odds ratio [OR] = 1.41, 95% confidence interval [CI]:1.17–1.70), whereas a negative association was observed between maternal TG, TC, LDL-C levels, and risk of SGA. Additionally, mothers with higher HDL-C levels were less likely to give birth to an LGA infant (OR=0.58, 95% CI:0.39–0.85). Importantly, associations between TG, TC, LDL-c, and SGA as well as between TG and LGA were primarily observed among pregnant women with insufficient vitamin D levels. As for HDL-C, the risk of LGA was lower in mothers with sufficient vitamin D (OR = 0.42, 95% CI:0.18–0.98) compared to those with insufficient vitamin D (OR = 0.65, 95% CI:0.42–0.99).

**Conclusion:**

Vitamin D status during the second trimester exerts a modifying effect on the association between lipid profiles and the risk of LGA and SGA infants.

## 1 Introduction

Adverse birth outcomes, including preterm birth (PTB), low birth weight (LBW), macrosomia, large for gestational age (LGA), and small for gestational age (SGA), have been identified as predictors of morbidity and mortality, as well as long-term health risks, such as metabolic syndrome, type II diabetes, and asthma (1–3). Numerous maternal factors, including gestational weight gain, pre-pregnancy body mass index (pre-BMI), and nutritional status during pregnancy, have been shown to be associated with adverse birth outcomes (4–6). Therefore, investigating the regulatory mechanisms underlying these outcomes during pregnancy is crucial for identifying potential preventive strategies.

During pregnancy, women undergo unique physiological changes and require increased nutrition and energy to support maternal metabolism and fetal growth. Maternal lipid profiles, including total cholesterol (TC), triglyceride (TG), low-density lipoprotein cholesterol (LDL-C), and high-density lipoprotein cholesterol (HDL-C), play crucial roles in providing energy for placental development (7). However, studies on the relationship between maternal dyslipidemia and adverse birth outcomes have yielded inconsistent results. For instance, a prospective study found a positive association between maternal TG levels and the risk of LGA infants, independent of maternal pre-BMI (8). Conversely, a cross-sectional analysis conducted in Brazil did not find a significant association between lipid intake and LGA newborns (9).

Vitamin D deficiency is a global public health problem affecting various age groups, particularly in pregnant women. Emerging evidence on the physiological activities of vitamin D has highlighted its role in reducing hepatic TG synthesis, cholesterol conversion, and the promotion of fatty acid (FA) oxidation (10, 11). The expression of coenzyme A reductase (HMG CoA reductase), sterol regulatory element binding proteins (SREBPs), and peroxisome proliferators activated receptor (PPAR) regulated by vitamin D might account for the improvements of lipid profile *in vivo* and *in vitro* (12). The optimal level of vitamin D for pregnancy health was unclear, but a higher risk of adverse pregnancy outcomes is more likely to be related to vitamin D deficiency. The serum vitamin D levels have been found to correlate with profile levels, which are attributed to the increased metabolic demands of pregnancy (13). Furthermore, high vitamin D levels in the second trimester may improve the lipid profile and mitigate the elevation of C-reactive protein induced by hyperlipidemia (14). A meta-analysis suggested an inverse association between maternal vitamin D levels and the risk of LBW, PTB, and SGA (15). The adequate vitamin D status during pregnancy has been considered a protective factor against SGA and is associated with improved infant growth (16).

Abnormal lipid profiles in pregnant women are considered as risk factors for LGA or SGA infants, but the effects of vitamin D status on these relationships remain unclear. Therefore, this study was performed to investigate the association between vitamin D status, lipid profile during the second mid-pregnancy, and the risk of LGA or SGA infants in Chinese women.

## 2 Materials and methods

### 2.1 Study population

**Figure 1:**
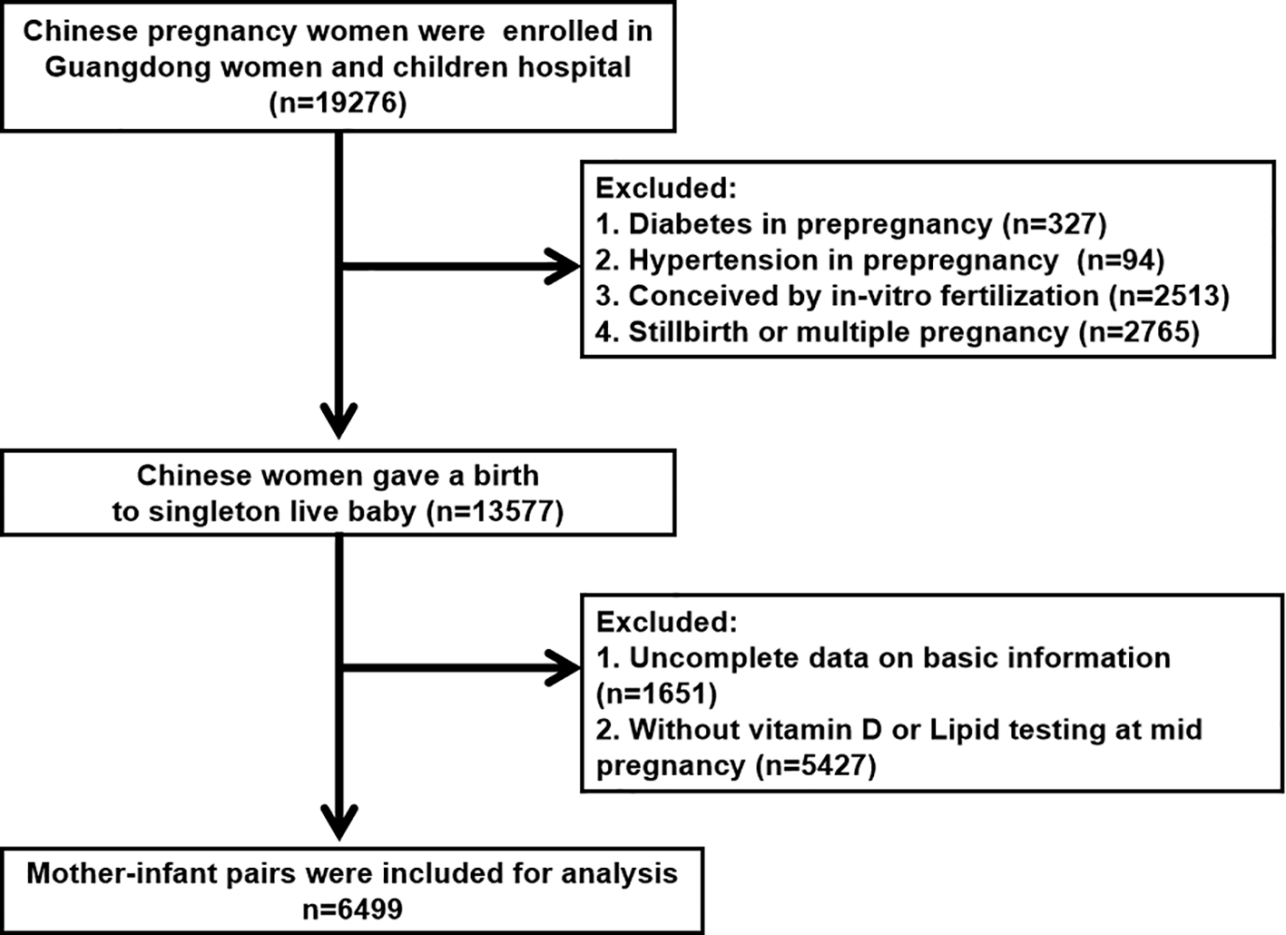
Flow chart for screening eligible participants.

This retrospective study included pregnant Chinese women who received prenatal care and intended to give birth at the Guangdong Women and Children’s Hospital (Guangzhou, China) between January 2020 and December 2021. This study was approved by the Ethics Committee of the Guangdong Women and Children’s Hospital (reference number 202301269). All participants were provided detailed information about the study and provided written informed consent. Women who met any of the following criteria were excluded from the study: (1) multiple pregnancies or stillbirths (n = 2765), (2) preexisting diabetes (n = 327), (3) preexisting hypertension (n = 94), (4) *in vitro* fertilization (n = 2513), or (5) incomplete data on basic information or testing (n = 1651) (**Figure 1**). Ultimately, 6499 mother-fetus pairs were included in this study.

### 2.2 Measurement for lipid profiles and vitamin D in mid-pregnancy

Non-fasting plasma samples were obtained during mid-pregnancy by a trained nurse (median 17.43 weeks of gestation, 90% range [14.14 to 24.86]). The concentrations of serum total cholesterol (TC), triglycerides (TG), low-density lipoprotein cholesterol (LDL-C), and high-density lipoprotein cholesterol (HDL-C) were analyzed using an automatic analyzer (Beckman Coulter, Brea, CA, USA) and a commercial kit (Leadman, Beijing, China). The vitamin D concentration was determined using an electrochemiluminescence immunoassay (Abbott Laboratories, IL, USA). Internal quality and quality control measurements were performed for each batch of analyses, with inter- and intra-assay coefficients of variation (CVs) below 10%.

### 2.3 Birth outcome and covariates

Anthropometric data on infants and basic information on mothers were obtained from the medical records of the study hospital. Immediately after birth, obstetric nurses recorded the birth weight, length, and head circumference of newborns. Data on maternal age, gravidity, parity, education level, smoking and drinking status, pregnancy complications (gestational diabetes mellitus [GDM] and hypertensive disorders in pregnancy [HDP]), gestational age at lipid profile testing, and the season of vitamin D measurement were extracted from medical records as potential covariates. Seasons of vitamin D measurement were defined as winter (December, January, February), spring (March, April, May), summer (June, July, August) and fall (September, October, November). We adjusted for potential covariates in the regression models based on previous reports. The maternal vitamin D status was defined according to the Endocrine Society’s Clinical Guidelines, with 25(OH)D levels below 75 nmol/L classified as non-sufficiency and levels equal to or above 75 nmol/L classified as sufficiency (17).

Newborns were classified as appropriate for gestational age (AGA), small for gestational age (SGA), or large for gestational age (LGA) based on Neonatal Birth Weight for Gestational Age and Percentile in 23 Cities of China. LGA was defined as birth weight above the 90th percentile, SGA as birth weight below the 10th percentile, and AGA as birth weight between the 10th and 90th percentiles (18).

### 2.4 Statistical analyses

Descriptive statistics were used to summarize the baseline data of the study participants. Continuous variables are reported as mean (standard deviation, SD) or median (interquartile range, IQR), while categorical variables are expressed as percentages. Non-parametric tests were used to compare continuous variables, and chi-square tests were used to compare categorical variables.

The Shapiro Wilk normality test was performed to verify the distribution of vitamin D and lipid profiles, which were right-skewed. To achieve a normal distribution, the raw values were log2-transformed. Spearman’s correlation coefficients (rs) were calculated to analyze the correlations between the log2-transformed concentrations of vitamin D and lipids.

Multivariate linear and logistic regression analyses were conducted to evaluate the association between serum lipid profiles and vitamin D concentration or status during mid-pregnancy. For LGA and SGA infants, multiple logistic regression analyses were performed to estimate the odds ratios (ORs) and 95% confidence intervals (CIs) based on TC, TG, HDL-C, LDL-C, and vitamin D concentrations. Pregnant women of normal weight served as the reference group. Regression models included potential covariates based on relevant reports. Subgroup analyses were conducted according to the maternal vitamin D status. Furthermore, the combined effects of vitamin D status and lipid concentration (TC, TG, HDL-C, and LDL-C) in the second trimester on LGA and SGA infants were investigated by adding the product interaction term of vitamin D status × lipid concentration to the models. A p-value for interaction less than 0.15 was used as a cutoff to explore the potential effect modification through stratification (19, 20).

All statistical analyses were performed using SPSS (version 26.0; SPSS, Chicago, IL, USA) and R version 3.3.3 (R Foundation for Statistical Computing). Statistical significance was defined as p < 0.05.

## 3 Results

A total of 6499 mother-infant pairs were included in this study, and their detailed demographic characteristics are presented in **Table 1**. Of the participants, the average age was 30.15 ± 4.30 years, 2947 (45.3%) were nulliparous, and 2345 (36.1%) underwent cesarean section. Only one woman had a history of smoking, and none of them smoked during pregnancy. Moreover, 4376 (67.3%) had a college degree or higher. The mean gestational age at lipids testing were 18.56 ± 3.82. Additionally, 5.3% (n = 342) of mothers experienced hypertensive disorders of pregnancy, and 17.9% (n = 1163) were diagnosed with gestational diabetes mellitus. The seasonal distribution of vitamin D testing in the second trimester was nearly equal between Fall and Winter, whereas spring had the highest percentage (34.8%). Additionally, there was seasonal variation in serum 25(OH)D concentration in this study. Maternal 25(OH)D was highest in summer (62.12 ± 22.81 nmol/L, n = 1604) followed by autumn (60.15 ± 21.23 nmol/L, n = 1243), winter (54.17 ± 21.78 nmol/L, n = 1390) and spring (54.04 ± 21.54 nmol/L, n = 2262), respectively (**Supplementary Figure S1**). Among the infants, 52.8% (n = 3429) were male. The mean birth weight, length, and gestational age at birth for the infants were 3.19 ± 0.43 kg, 49.46 ± 1.93 cm, and 39.23 ± 1.42 weeks, respectively.

**Table 1:** Clinical data of the study population.

Characteristics	Mean ± SD or n (%)
Maternal age (years)	30.15 ± 4.30
parity	
Multiparous	3552 (54.7%)
Nulliparous	2947 (45.3%)
Education level	
College	4376 (67.3%)
High School	1019 (15.7%)
< High School	1104 (17%)
Cesarean section	2345 (36.1%)
Pre-pregnancy BMI (kg/m^2^)	21.04 ± 4.83
BMI status	
Underweight	4348 (66.9%)
Normalweight	1262 (19.4%)
Overweight	717 (11%)
Obesity	172 (2.6%)
GWG	13.74 ± 4.56
GDM	1163 (17.9%)
HDP	342 (5.3%)
PTB	310 (4.8%)
Season at Vitamin D testing	
Spring	2262 (34.8%)
Summer	1604 (24.7%)
Autumn	1243 (19.1%)
Winter	1390 (21.4%)
Gestational age at lipid testing	18.56 ± 3.82
Neonatal characteristics	
Boys	3429 (52.8%)
SGA	635 (9.8%)
LGA	447 (6.9%)
Birth weight (kg)	3.19 ± 0.43
Length (cm)	33.52 ± 1.35
Head (cm)	49.46 ± 1.93
Gestational age (weeks)	39.23 ± 1.42

SD, standard deviation; BMI, body mass index; GWG, Gestational weight gain; HDP, hypertensive disorders of pregnancy; GDM, gestational diabetes mellitus; SGA, small for gestational age; LGA, large for gestational age.

The median (25th–75th) values of the four lipid parameters in the second trimester were as follows: 1.83 (1.32–2.15) mmol/L for TG, 5.61 (4.85–6.24) mmol/L for TC, 1.89 (1.65–2.10) mmol/L for HDL-C, and 3.09 (2.56–3.56) mmol/L for LDL-C (**Table 2**). The overall range of vitamin D concentrations was 10.5–159.2 nmol/L, with a mean ± SD of 57.23 ± 22.14 nmol/L. 20.7% of the patients (n=1350) were classified into the sufficient vitamin D group. Women with sufficient vitamin D in mid pregnancy have higher cholesterol levels than those with non-sufficient vitamin D (**Supplementary Table S1**). Similar findings were also observed in Spearman correlation analysis, it is suggested that vitamin D concentrations was correlated with cholesterol levels except for TG (**Supplementary Table S2**).

**Table 2:** Distributions of maternal vitamin D and lipid profiles in the second trimester.

Analytes	Mean	GM	Percentiles		
			25	50	75
TG	1.83	1.69	1.32	1.67	2.15
TC	5.61	5.51	4.85	5.50	6.24
HDL-C	1.89	1.86	1.65	1.87	2.10
LDL-C	3.09	2.99	2.56	3.04	3.56
vitamin D	57.23	52.83	40.3	55.4	71.9

TC, total cholesterol; TG, triglycerides; LDL-C, low-density lipoprotein cholesterol; HDL-C, high-density lipoprotein cholesterol; GM, Geometric Mean.

**Figure 2:**
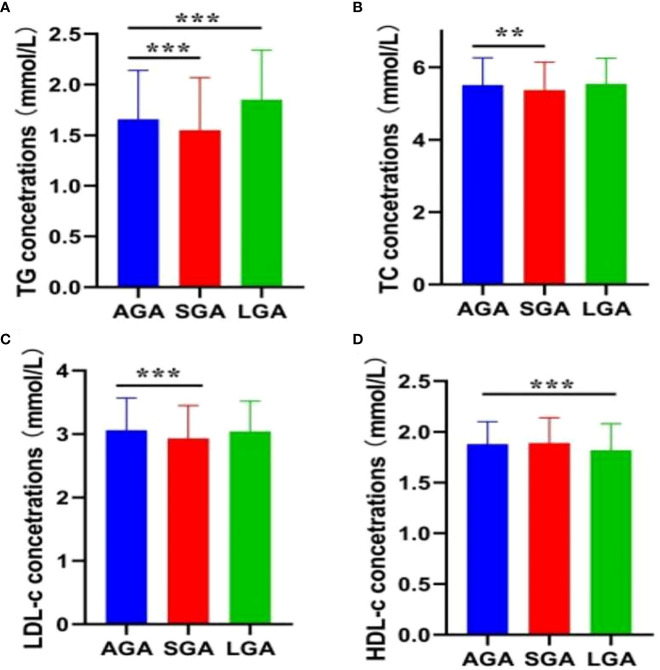
Maternal lipid profile in mid-pregnancy and fetal growth. **(A)** TG; **(B)** TC; **(C)** LDL-C; **(D)** HDL-C. Error bars are presented as mean (SD) for continuous variables with a normal distribution, or as median (90% range) for continuous variables with a skewed distribution. **P < 0.01; ***P < 0.001.

The prevalence of LGA and SGA infants was 6.9% (n = 447) and 9.8% (n = 635), respectively. **Figure 2** presents the lipid profiles and vitamin D concentrations in the AGA, SGA, and LGA groups. Women with infants born LGA exhibited higher levels of TG and LDL-C compared to women with infants born AGA. Conversely, the TG, TC, and LDL-C levels were significantly lower compared than in the control group (p < 0.05) in the SGA group. In addition, women with a LGA newborn had lower levels of HDL-C. **Table 3** presents the association between maternal lipid parameters in the second trimester and the risk of LGA or SGA. TG levels were positively associated with the risk of LGA (OR=1.41, 95% CI 1.17–1.70, p < 0.001), while maternal TG, TC, and LDL-C were negatively associated with the risk of SGA. Additionally, higher HDL-C levels in mothers were associated with a lower likelihood of delivering an LGA infant (OR = 0.58, 95% CI 0.39–0.85). No significant association was found between the vitamin D status and the risk of LGA or SGA infants (all p > 0.05).

**Table 3:** The association of maternal lipid profile concentrations and vitamin D categories with LGA or SGA in early pregnancy in the second trimester.

	AGA (n=5417)	SGA (n=635) OR (95% CI)	p	LGA (n=447) OR (95% CI)	p
Lipids^a^					
TG	reference	0.74 (0.62–0.88)	0.001	1.41 (1.17–1.70)	0.000
TC	reference	0.65 (0.46–0.90)	0.01	0.95 (0.65–1.39)	0.786
HDL-C	reference	1.10 (0.79–1.54)	0.563	0.58 (0.39–0.85)	0.005
LDL-C	reference	0.75(0.59–0.94)	0.013	0.98 (0.75–1.30)	0.905
Vitamin D Binary ^b^					
Sufficient group		reference		reference	
Non-Sufficient group		1.20 (0.96–1.49)	0.107	1.02 (0.80–1.31)	0.847
Vitamin D ^b^ (10.9–159.2nmol/L)	reference	0.92 (0.80–1.07)	0.274	0.98 (0.83–1.17)	0.850

Maternal Vitamin D and lipid profiles were log2-transformed in the model

a The models were adjusted for education, maternal age, parity, delivery mode, infant sex, HDP, GDM, pre-BMI, and gestational age at lipid testing.

b The models were adjusted for education, maternal age, parity, delivery mode, infant sex, HDP, GDM, pre-BMI, and season of vitamin D testing.

The effect of vitamin D on the association between lipid profiles and risk of LGA or SGA was explored by dividing the study population into two different vitamin D categories. Although no interaction effect was observed among these birth outcomes (p for interaction > 0.15), the effect of the lipid profile differed because of the vitamin D status (**Table 4**). For TG, mothers in the vitamin D non-sufficiency group with higher TG level was related to an increased risk (OR=1.40, 95% CI:1.13–1.74) for LGA. Regarding cholesterol, no associations were found between the HDL-C and SGA levels in this subgroup analysis. Nonetheless, HDL-C levels were negatively associated with the risk of LGA infants regardless of the vitamin D status (OR=0.65 in pregnant women with insufficient vitamin D; OR=0.42 in the sufficient vitamin D group). Furthermore, when the population was stratified by vitamin D categories, higher levels of TC and LDL-C were associated with a decreased risk of SGA (TC: OR=0.65, 95% CI: 0.46–0.94; LDL-C: OR=0.74, 95% CI: 0.57–0.95) among pregnant women in the non-sufficient vitamin D group.

**Table 4:** Associations between maternal lipid levels in second trimester and risk of LGA or SGA in multinomial logistic regression models, stratified by vitamin D level.

	Vitamin D status		*p* for interaction
	Non- Sufficient group	Sufficient group	
TG			
AGA	reference	reference	
SGA	0.77 (0.63–0.93)**	0.66 (0.43–1.02)	0.821
LGA	1.40 (1.13–1.74)**	1.31 (0.85–2.03)	0.416
TC			
AGA	reference	reference	
SGA	0.65 (0.46–0.94)*	0.65 (0.29–1.43)	0.859
LGA	0.96 (0.62–1.48)	0.96 (0.40–2.30)	0.487
HDL-C			
AGA	reference	reference	
SGA	1.16 (0.81–1.68)	0.82 (0.37–1.80)	0.228
LGA	0.65 (0.42–0.99)*	0.42 (0.18–0.98)*	0.300
LDL-C			
AGA	reference	reference	
SGA	0.74 (0.57–0.95)*	0.82 (0.47–1.44)	0.749
LGA	0.92 (0.68–1.25)	1.36 (0.71–2.61)	0.726

Maternal Vitamin D and lipid profiles were log2-transformed in the model

The models were adjusted for education, maternal age, parity, delivery mode, infant sex, HDP, GDM, pre-BMI, Season at vitamin D testing, and gestational age at lipid testing.

P for interaction was assessed by likelihood ratio test.

*P < 0.05; **P < 0.01.

## 4 Discussion

In this retrospective study, the prevalence rates of SGA and LGA in pregnant Chinese women were 9.8% and 6.9%, respectively. Only 20.5% of the participants (n=1350) demonstrated sufficient vitamin D levels during their second trimester. The TG levels during mid-pregnancy were positively associated with an increased risk of LGA infants, whereas HDL-C levels were negatively correlated with LGA risk. Maternal TG, TC, and LDL-C levels were associated with a decreased risk of being SGA; however, no significant association was observed for HDL-C. Although no significant interaction effects were identified, notable differences were observed in the subgroup analysis. Our findings suggest that TG, TC, and LDL-C levels are positively correlated with decreased odds of being SGA among pregnant women with insufficient vitamin D levels. Notably, mothers with sufficient vitamin D levels had a significantly lower risk of LGA infants than those with insufficient vitamin D levels.

Risk of LGA or SGA are associated with maternal conditions, such as maternal dietary intake, obesity, metabolic changes, genetic polymorphisms, environmental factors, and gestational weight gain. For example, we have reported that pregnant women with lower gestational weight gain and MTHFR A1298C AA genotype were more likely to experience SGA (21). A prospective multi-racial/ethnic cohort study suggested that pregnant women with poorer maternal diet in early pregnancy were more likely to have an LGA infant, even after adjustment for maternal obesity (22). As an important indicator for lipid metabolism, maternal lipid profiles are related to overnutrition and increased throughout pregnancy. This suggests that lipid profiles have play an important role in fetal growth. It was reported that higher TG levels in early pregnancy are associated with increased embryonic size, fetal head circumference, and overall growth rates (8). The pathway of TG from maternal circulation into the placenta to support fetal growth is complex because it cannot cross the placenta. Fatty acid hydrolyzed from TG can enter fetal circulation through placental trophoblasts and provided energy for the growth of fetus (23). However, hyperlipidemia can lead to adverse pregnancy complications and perinatal outcomes, potentially affecting offspring development (24, 25). In this study, we suggested a positive association between the maternal TG levels in the second trimester and the risk of LGA (OR=1.41, 95% CI=1.17–1.70), as well as a negative association between maternal TG levels and the risk of SGA (OR=0.74, 95% CI=0.62–0.88). The differential TG levels observed in our study may explain these results, as TG levels were higher in mothers of LGA infants and lower in mothers of SGA infants. Compared with normal-weight controls, we found that TG concentrations were higher in women born to LGA infants and lower in mothers with SGA infants. Maternal cholesterol is important for membrane function and development of the fetus. Recent studies have suggested that maternal TC and LDL-C levels are valuable markers of abnormal fetal development. Serizawa et al. demonstrated that lower maternal LDL-C levels in the second trimester were associated with an increased risk of delivering an SGA infant at term (26). Chen et al. reported a negative association between second trimester TC and LDL-C levels and SGA (27). Consistent with these results, our analysis showed a negative association between TC or LDL-C concentrations and the risk of SGA infants (OR=0.65 TC, OR=0.75 LDL-C). HDL-C plays an important role in cholesterol homeostasis by maintaining a favorable sterol balance in extraembryonic fetal tissues to support fetal growth and development (28). For instance, an increase of 10 mg/dL in HDL-C from preconception to 28 weeks was associated with decreased odds of LGA (OR = 0.63, 95% CI: 0.46–0.86), with a stronger association observed in women with a pre-pregnancy BMI over 25 (29). In our study, pregnant women who delivered LGA newborns had lower HDL-C levels than those who delivered AGA newborns, which is consistent with the findings of a study involving 549 pregnant Chinese women (30). Furthermore, our results indicated that higher HDL-C levels in mothers were associated with a reduced risk of LGA infants (OR=0.58), even after adjusting for pre-BMI and GDM. However, a prospective study proposed a negative association between HDL-C levels in early pregnancy and LGA, and these effects may become non-significant after adjusting for pre-pregnancy BMI and early pregnancy maternal glucose levels (8). The inconsistent results observed across studies may be attributed to differences in population settings, confounding variables, and timing of measurements. In addition, the ethnic differences and genetic factors might also modify the associations between maternal cholesterol and birth weight (31, 32). Future multiple-ethnic studies must investigate the effect of genetic differences on the relationship between maternal lipid metabolism and fetal development.

Vitamin D, a fat-soluble vitamin biosynthesized via an ultraviolet radiation-mediated process or absorbed from dietary sources, plays a crucial role in the calcium phosphate metabolism and bone construction. In this analysis, we found that the maternal 25(OH)D concentration in second trimester were highest in summer (62.12 ± 22.81 nmol/L) and lowest in spring (54.04 ± 21.78 nmol/L). During pregnancy, low vitamin D concentrations are commonly observed in pregnant women due to the increased physiological demand for vitamin D. In this study, the mean vitamin D concentration was 57.23 nmol/L, which is similar with a retrospective cohort study conducted in Guangzhou. They reported that pregnant women exhibited an average vitamin D level of 59.3 nmol/L (33). The prevalence of insufficient vitamin D (< 75 nmol/L) was 79.2% (n = 5149), which was nearly four times higher than that in the sufficient vitamin D group. These findings are consistent with a prospective observational study conducted in Guangzhou, which reported a 67.5% prevalence of insufficient vitamin D among pregnant women (34). Similar results were observed in pregnant women from Brazil (69%), Kenya (74.4%), and rural Bangladesh (64.5%) (35–37). Although increased studies have shown that vitamin D deficiency in serum during pregnancy is closely related to a series of adverse pregnancy outcomes (38, 39), our study suggests that the vitamin D status at mid-pregnancy, even in the vitamin D-deficient group, was not associated with LGA or SGA.

Several studies have shown that the vitamin D status may be related to improvements in lipid profiles. For example, a prospective birth cohort study of 6714 pregnant women in Hefei (another city in China) suggested that increased serum vitamin D levels were significantly associated with decreased maternal TC, TG, HDL-C, and LDL-C levels in the second trimester (14). Sharif-Askari et al. found that vitamin D deficiency was associated with HDL-C dyslipidemia in insulin-resistant individuals (40). Pregnant women with sufficient vitamin D have higher cholesterol levels (TC, HDL-C, and LDL-C) than those with non-sufficient vitamin D in our study population. This may be because vitamin D and cholesterol metabolism share a similar biosynthetic pathway. Additionally, vitamin D exerts a potent anti-lipolytic action, increases the intracellular calcium levels, regulates the renin-angiotensin system, and suppresses lipolysis in human adipocytes (41). Vitamin D can directly and indirectly influence lipid levels through its effects on serum parathyroid hormone (PTH) and calcium balance, thereby regulating lipid metabolism (42). However, there is no consensus on the association between vitamin D and lipid metabolism. In zebrafish model, vitamin D was reported to reduce the deposition of lipid via regulation of mitochondrial biogenesis (11). Considering the effect of vitamin D on fat storage and lipid metabolism, we hypothesized that vitamin D may have a modifying effect on the association between lipid levels and LGA and SGA. In the subgroup analysis, the effects of TG, TC, and LDL-C on LGA or SGA risks were only observed in the vitamin D insufficient group. Furthermore, a higher HDL-C level was associated with a lower likelihood of giving birth to an LGA infant among pregnant women with sufficient vitamin D levels in the second trimester (OR = 0.42) than among those with insufficient vitamin D levels (OR = 0.65). The vitamin D status in a sufficient status appears to have a beneficial effect in reducing the serum TC, LDL-C, and TG levels (43). Although the effect of dietary intake did not evaluate on the level of vitamin D and lipid profile in the present study, our results suggest that the vitamin D status at mid-pregnancy may modify the association between the lipid profile and risk of LGA or SGA.

In this study, we conducted a comprehensive investigation involving 6499 mother-infant pairs to assess the association between vitamin D levels, lipid profiles in the second trimester, and the occurrence of SGA or LGA. Additionally, we explored the potential effect of vitamin D status on the association between maternal lipid metabolism and risk of SGA or LGA. Our findings suggest that pregnant women with abnormal lipid profiles should be monitored for their vitamin D status to mitigate the risk of SGA or LGA. However, it is important to acknowledge the limitations of this study. First, we collected serum samples during the second trimester, although it is recommended to collect maternal lipid concentrations throughout pregnancy and before conception. Second, our analysis did not account for various potential confounding factors, such as eating patterns, vitamin D supplementary, physical activity, and other environmental exposures, which may have influenced the reliability of our results. Further investigations with larger sample sizes, diverse populations, and prospective study designs are necessary to validate the association between maternal vitamin D levels and subsequent delivery outcomes.

## 5 Conclusion

In summary, our retrospective study, based on a Chinese population encompassing 6499 mother-infant pairs, examined the relationship between vitamin D levels, lipid profiles, and the risk of SGA or LGA. We observed a significant association between vitamin D and cholesterol levels during mid-pregnancy. Moreover, our findings provide evidence that the vitamin D status may modify the association between HDL-C levels and the risk of LGA. These results could serve as guidelines for managing lipid profiles and nutritional interventions during pregnancy to improve birth outcomes in Chinese populations. However, further investigations with larger sample sizes, diverse populations, and prospective or multicenter designs are warranted to confirm and expand upon our findings.

## Data availability statement

The raw data supporting the conclusions of this article will be made available by the authors, without undue reservation.

## Ethics statement

The studies involving humans were approved by the Ethics Committee of the Guangdong Women and Children’s Hospital. The studies were conducted in accordance with the local legislation and institutional requirements. Written informed consent for participation was not required from the participants or the participants’ legal guardians/next of kin in accordance with the national legislation and institutional requirements.

## Author contributions

XHZ: Data curation, Formal analysis, Investigation, Methodology, Resources, Writing – original draft. KL: Data curation, Investigation, Software, Writing – original draft. CL: Investigation, Methodology, Project administration, Writing – original draft. YC: Conceptualization, Data curation, Methodology, Writing – original draft. XDZ: Data curation, Investigation, Methodology, Validation, Writing – original draft. WW: Formal analysis, Software, Supervision, Writing – original draft. ML: Conceptualization, Supervision, Writing – review & editing. CG: Conceptualization, Funding acquisition, Project administration, Supervision, Writing – review & editing.
