# Supplementary material for: Association between maternal lipid profiles and vitamin D status in second trimester and risk of LGA or SGA: a retrospective study
Source: Front Endocrinol (Lausanne). 2024 Jul 1;15:1297373. doi: 10.3389/fendo.2024.1297373 (PMC11246877; doi:10.3389/fendo.2024.1297373)
Supplement: Supplementary file 1 [file DataSheet_1.docx]

**Supplementary Material**

**Association between maternal lipid profiles and vitamin D status in second trimester and risk of LGA or SGA: a retrospective study**

Xianhua Zheng^1^†, Kefeng Lai^1^†, Chengyi Liu^1^, Yuan Chen^1^, Xiandan Zhang^1^, Weixiang Wu^1^, Mingyong Luo^1^, Chunming Gu^1^*

1Department of Clinical Laboratory, Guangdong Women and Children Hospital, Guangzhou, China

* Correspondence:

Chunming Gu, [guchunming0119@163.com](mailto:guchunming0119@163.com)

†These authors have contributed equally to this work.

Number of pages: 3;

Number of tables: 2;

Number of figure: 1;

| Table S1. Clinical data of the study population with different vitamin D status. | | | |
| --- | --- | --- | --- |
| Characteristics | Vitamin D  (<75 mmol/L, n=5149) | Vitamin D  (≥75 mmol/L, n=1350) | *p* value |
| Maternal age (years) | 29.92 ± 4.27 | 31.03 ± 4.31 | < 0.001 |
| parity, n (%) |  |  | 0.155 |
| Multiparous | 2791 (42.9%) | 761 (11.7%) |  |
| Nulliparous | 2358 (36.3%) | 589 (9.1%) |  |
| Edu, n (%) |  |  | 0.399 |
| College | 3469 (53.4%) | 901 (14.0%) |  |
| High School | 860 (13.2%) | 244 (3.8%) |  |
| < High School | 820 (12.6%) | 199 (3.1%) |  |
| Cesarean section | 1868 (28.7%) | 477 (7.3%) | 0.520 |
| Pre-pregnancy BMI (kg/m^2^) | 21.06 ± 4.64 | 20.97 ± 5.48 | 0.561 |
| GWG | 16.21 ± 122.67 | 13.60 ± 4.70 | 0.437 |
| GDM, n (%) | 941 (14.5%) | 222 (3.4%) | 0.118 |
| HDP, n (%) | 279 (4.3%) | 63 (1%) | 0.271 |
| Season at Vitamin D testing |  |  | < 0.001 |
| Spring | 1886 (29%) | 376 (5.8%) |  |
| Summer | 1162 (17.9%) | 442 (6.8%) |  |
| Autumn | 948 (14.6%) | 295 (4.5%) |  |
| Winter | 1153 (17.7%) | 237 (3.6%) |  |
| Gestational age at lipid testing | 18.37 ± 3.77 | 19.30 ± 3.92 | < 0.001 |
| Neonatal characteristics |  |  |  |
| Boys | 2691 (41.4%) | 738 (11.4%) | 0.115 |
| Birth weight (kg) | 3.19 ± 0.43 | 3.21 ± 0.43 | 0.152 |
| Gestational age | 39.23 ± 1.44 | 39.22 ± 1.37 | 0.746 |
| Length (cm) | 33.52 ± 1.36 | 33.52 ± 1.29 | 0.939 |
| Head (cm) | 49.45 ± 1.94 | 49.50 ± 1.91 | 0.449 |
| TG, mean ± sd | 1.83 ± 0.81 | 1.80 ± 0.69 | 0.103 |
| TC | 5.59 ± 1.09 | 5.70 ± 1.04 | 0.003 |
| HDL-c | 1.88 ± 0.34 | 1.92 ± 0.33 | < 0.001 |
| LDL-c | 3.08 ± 0.80 | 3.14 ± 0.76 | 0.011 |
| Vitamin D | 48.75 ± 15.08 | 89.59 ± 13.14 | < 0.001 |

| Table S2. Spearman correlations between individuals for maternal lipid profiles and vitamin D levels measured in second trimester. | | | | |
| --- | --- | --- | --- | --- |
|  | Vitamin D | TG | TC | HDL-C |
| TG | 0.008 |  |  |  |
| TC | 0.055** | 0.326** |  |  |
| HDL-C | 0.071** | -0.067** | 0.531** |  |
| LDL-C | 0.047** | 0.323** | 0.873** | 0.277** |


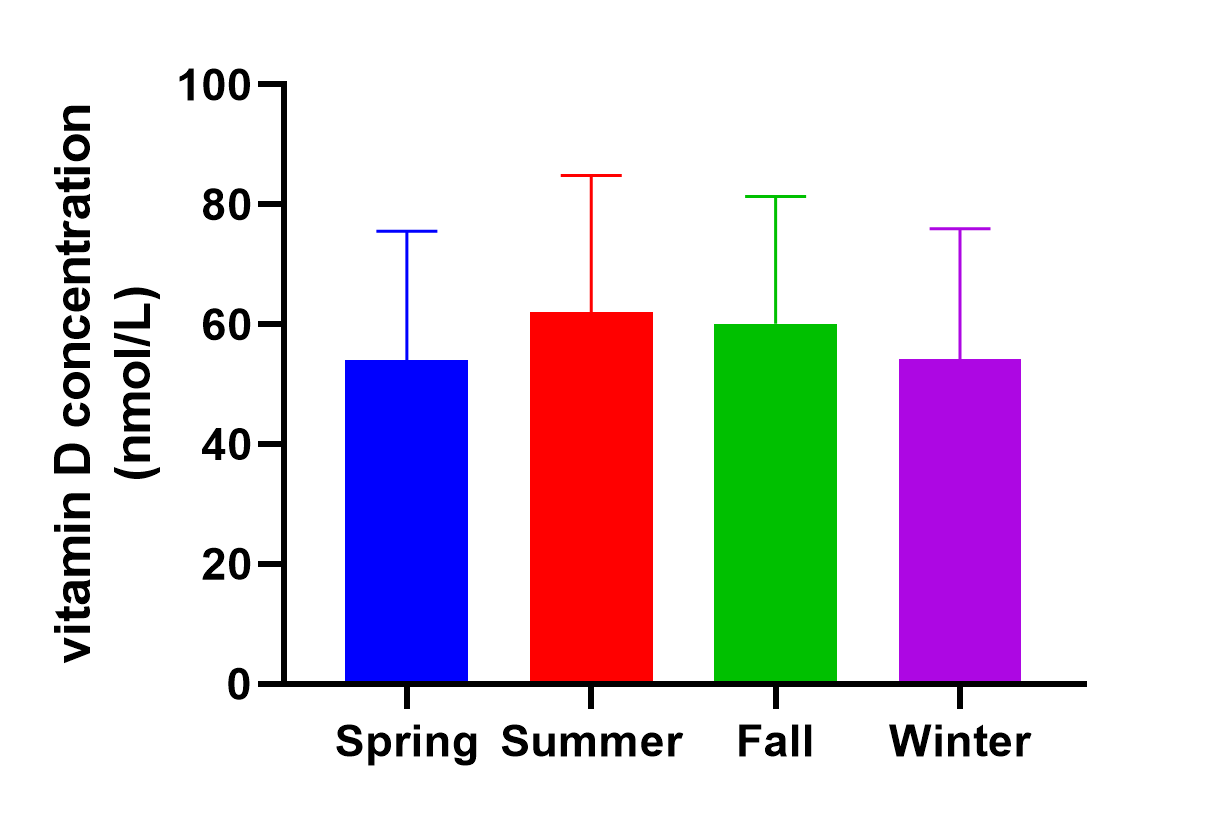


Figure S1. The concentration of 25(OH)D in the serum during the different seasons.
